# Effects of acupuncture treatment for myasthenia gravis: A systematic review and meta-analysis

**DOI:** 10.1371/journal.pone.0291685

**Published:** 2024-01-02

**Authors:** Hua Xue, Li Zeng, Hongxian He, Dongxun Xu, Kaixin Ren

**Affiliations:** 1 Department of Neurology, Sichuan Taikang Hospital, Chengdu, Sichuan, China; 2 Department of Respiratory, Affiliated Hospital of Youjiang Medical University for Nationalities, Baise, Guangxi, China; 3 Department of Rehabilitation, Sichuan Taikang Hospital, Chengdu, Sichuan, China; 4 Affiliated Hospital of Yunnan University, Kunming, Yunnan, China; The University of Mississippi Medical Center, UNITED STATES

## Abstract

**Background:**

Randomized controlled trials (RCTs) of acupuncture for myasthenia gravis (MG) were searched and the efficacy of acupuncture in the treatment of MG was evaluated by meta-analysis.

**Methods:**

We searched for RCTs in six main electronic databases, and collected RCTs of acupuncture treatment for MG from database creation to 28 February 2023. The main outcome was the effective rate and the secondary outcome was the Traditional Chinese Medicine (TCM) relative clinical score, absolute clinical score (ACS) of MG, Quantitive myasthenia gravis score (QMG), quality of life, and adverse events. Odds ratios (ORs) and weighted mean differences (WMD) and 95% confidence intervals (CI) were used to assess pooled effect estimates using Review Manager software.

**Results:**

A total of 14 RCTs were included. Meta-analysis showed that the effective rate in the acupuncture group was significantly improved compared with conventional Western medicine alone [OR = 4.28, 95% CI (2.95, 6, 22), P<0.005]. The pooled WMDs revealed that TCM relative clinical score [WMD = -2.22, 95% CI = (-2.53, -1.90), P<0.005], ACS of MG [WMD = -3.14, 95% CI = (-3.67, -2.62), P<0.005], and QMG [WMD = -0.88, 95% CI = (-1.46, -0.29), P<0.005] in the acupuncture group was lower than the control group. Adverse reactions related to acupuncture and quality of life were less mentioned among included RCTs.

**Conclusion:**

This meta-analysis demonstrated that acupuncture as an auxiliary may play a positive role in treating MG. It can improve the effective rate of treatment, and reduce TCM relative clinical score, ACS of MG, and QMG. However, the quality of included studies was generally low and caution should be exercised when considering this treatment option. In the future, more rigorous study designs and high-quality RCTs are needed to verify the efficacy of acupuncture in the treatment of MG, because the results of high-quality RCTs are more reliable and accurate.

## 1. Introduction

Myasthenia gravis (MG) is an acquired autoimmune disease caused by neuromuscular postsynaptic membrane damage and abnormal transmission function at the neuromuscular junction, resulting in muscle weakness [1, 2]. The pathogenesis of MG is mainly related to acetylcholine receptor mediated, cellular immune dependence and complement participation [3, 4]. The clinical manifestations of MG are fluctuating muscle weakness and fatigue of various muscle groups, and dyspnea may occur in severe cases [5]. The age of onset ranged from young to late adulthood, with the peak incidence in young adult women and elderly men [6]. The incidence is estimated at 10 to 25 per 100,000 and the worldwide prevalence at 700,000 [6, 7].

At present, conventional treatments of MG include: acetylcholinesterase inhibitors (AchEI), immunosuppressants, plasma exchange, thymectomy, etc [7, 8]. Pyridostigmine is one of the common AchEI for the treatment of MG. Its mechanism is to inhibit the activity of cholinesterase (AChE) by binding to AChE, which increases the concentration of acetylcholine (ACh) in the neuromuscular junction, thereby stimulating ACh receptor to improve the symptoms of muscle weakness [9]. Prednisone and tacrolimus are commonly used immunosuppressants. Studies have demonstrated that early use of immunosuppressants can improve the prognosis of MG patients, such as reducing the recurrence rate and improving the quality of life of patients [10]. However, these costly treatments cannot completely cure MG, and most patients are easy to appeared adverse reactions of drugs, such as diarrhea, nausea, vomiting, hydrostomia, and even muscle twitching and dependence [7, 8]. Traditional Chinese medicine (TCM) including traditional Chinese medicine decoction, Chinese patent medicine, acupuncture and other therapies have certain advantages in improving the efficiency of disease treatment, reducing the recurrence rate, reducing side effects and improving the condition [11]. For example, Shengyang Jutuo decoction, Buzhong Yiqi decoction, etc. are commonly used Chinese medicine decoction which alleviates symptoms by improving patient’s autoimmune status in China. As an important part of Chinese medicine, acupuncture has been commonly practiced treat various diseases in clinical practice in China [11]. As a non-pharmacological treatment, acupuncture rarely causes adverse effects such as gastrointestinal discomfort, liver function damage, and kidney function damage because it does not involve pharmacological metabolism and other effects. Acupuncture can dredge the meridians, regulate “Yin” and “Yang”, stimulate local nerves quickly and directly through physical action, and improve the local immune microenvironment, which has been confirmed in many animal experiments and clinical observations [12]. There are many types of acupuncture, such as fire acupuncture, electric acupuncture, manual acupuncture, warm acupuncture, water acupuncture, etc [13, 14]. These methods have less expensive and few side effects.

With the deepening of TCM treatment of MG, many studies have shown that acupuncture combined with drugs has therapeutic advantages in the treatment of MG [15]. So far, systematic reviews of acupuncture for MG are relatively scant, whereas most of the existing randomized control trials (RCTs) are limited by the small sample size and study design flaws, which may bring about controversial results and cannot provide adequate evidence for further clinical applications. Therefore, we conducted a systematic review and meta-analysis of published 12 RCTs using the PRISMA (Preferred Reporting Items for Systematic Reviews and Meta-Analyses) guidelines, aiming to evaluate the efficacy and safety of acupuncture in the treatment of MG, and provided reliable evidence-based medical evidence for the precise treatment of MG [16].

## 2. Material and methods

This meta-analysis (registration No. CRD42023402885) focused on RCTs involving acupuncture interventions on myasthenia gravis, which abided by the Preferred Reporting Items for Systematic Reviews and Meta-Analyses statement [17].

### 2.1 Eligibility criteria

Articles meeting the following criteria were included: (1) population: patients were diagnosed as myasthenia gravis according to WHO diagnostic criteria, which were based on typical clinical features of MG and any of the following three conditions, including neostigmine test, electrophysiological features, and serum anti-ACh antibody test; (2) interventions: patients in the experimental group received acupuncture therapy, including manual acupuncture, electronic acupuncture, and scalp acupuncture, or acupuncture therapy with medicine therapy; (3) control: patients in the control group received medicine therapy, placebo treatment such as sham acupuncture, or no treatment; (4) outcome measures: the main outcome focused on the effective rate. The clinical efficacy of MG was considered meeting one of the following conditions as effective: 1) Traditional Chinese medicine (TCM) relative clinical score decreased by more than 25% compared with the previous; 2) Improvement of symptoms. The effective rate was regarded as a dichotomous measure (effective or ineffective). [18] The secondary outcome measures included Traditional Chinese medicine (TCM) relative clinical score, absolute clinical score(ACS) of myasthenia gravis, Quantitive myasthenia gravis score (QMG), quality of life, and adverse events; (5) RCTs regardless of publication language were Chinese and English. The following were excluded from the analysis: non-RCTs, uncontrolled trials, protocols for RCTs and inappropriate intervention studies, such as comparisons between two groups focusing on different acupuncture methods or different acupuncture points.

### 2.2 Search strategy

From database creation to 28 February 2023, we searched the following six electronic databases: Cochrane Central Register of Controlled Trials (Central), PubMed, Embase, China National Knowledge Infrastructure (CNKI), China Biomedical Literature Database (CBM) and Wanfang Database. We manually searched the relevant literature to identify other potentially eligible studies. The keywords for literature retrieval were “acupuncture,” “acupuncture therapy,” “electroacupuncture,” “acupuncture treatment,” “myasthenia gravis,” and “randomized controlled trial.” The search terms and search strategy are presented in S1 Table.

### 2.3 Data collection

Microsoft Excel was used to compile electronic data extraction forms and manage information extracted from eligible articles, including country, year of publication, sample size unlimited, mean age of patients, interventions in the experimental group, interventions in the control group, and outcomes (include effective rate, TCM relative clinical score, ACS, QMG, quality of life, and adverse events). Investigators extracted and verified all data to ensure accuracy. Corresponding authors were contacted and asked to clarify any ambiguities and, if possible, submit missing information by phone or email.

### 2.4 Quality assessment

Two reviewers independently assessed the quality of the included trials using the Cochrane Collaboration tool to assess the risk of bias (ROB) which provides a rigorous independent assessment of different terms of study quality. The Cochrane Collaboration tool assesses seven important sources of bias including random sequence generation, allocation concealment, blinding of participants and personnel, blinding of outcome assessment, incomplete outcome data, selective reporting, and other. The reviewer’ s judgment for each item including “low risk”, “high risk” or “unclear”of bias. Any disagreements were settled by discussion with another reviewer.

### 2.5 Data synthesis and statistical methods

Data were analyzed using Cochrane Collaboration Meta-analysis software (Review Manager 5.4). Dichotomous data, such as effective rate was expressed by odds ratio (OR) or relative risk (RR) and their 95% confidence interval (CI). Continuous data such as TCM relative clinical score, ACS of MG, quantitive myasthenia gravis score were expressed by weighted mean difference (WMD) and its 95% CI, and P<0.05 was considered statistically significant. The heterogeneity test was carried out on the included literature. If P ≤0.1, I^2^ ≥ 50%, it indicated the existence of heterogeneity, and the random effect model was used for heterogeneity analysis. Statistical heterogeneity or less heterogeneity, using a fixed-effects model. The results of meta-analysis were expressed in forest plots, and funnel plots were used to evaluate publication bias if included articles more than 10.

## 3. Results

### 3.1 Study selection

**Fig 1:**
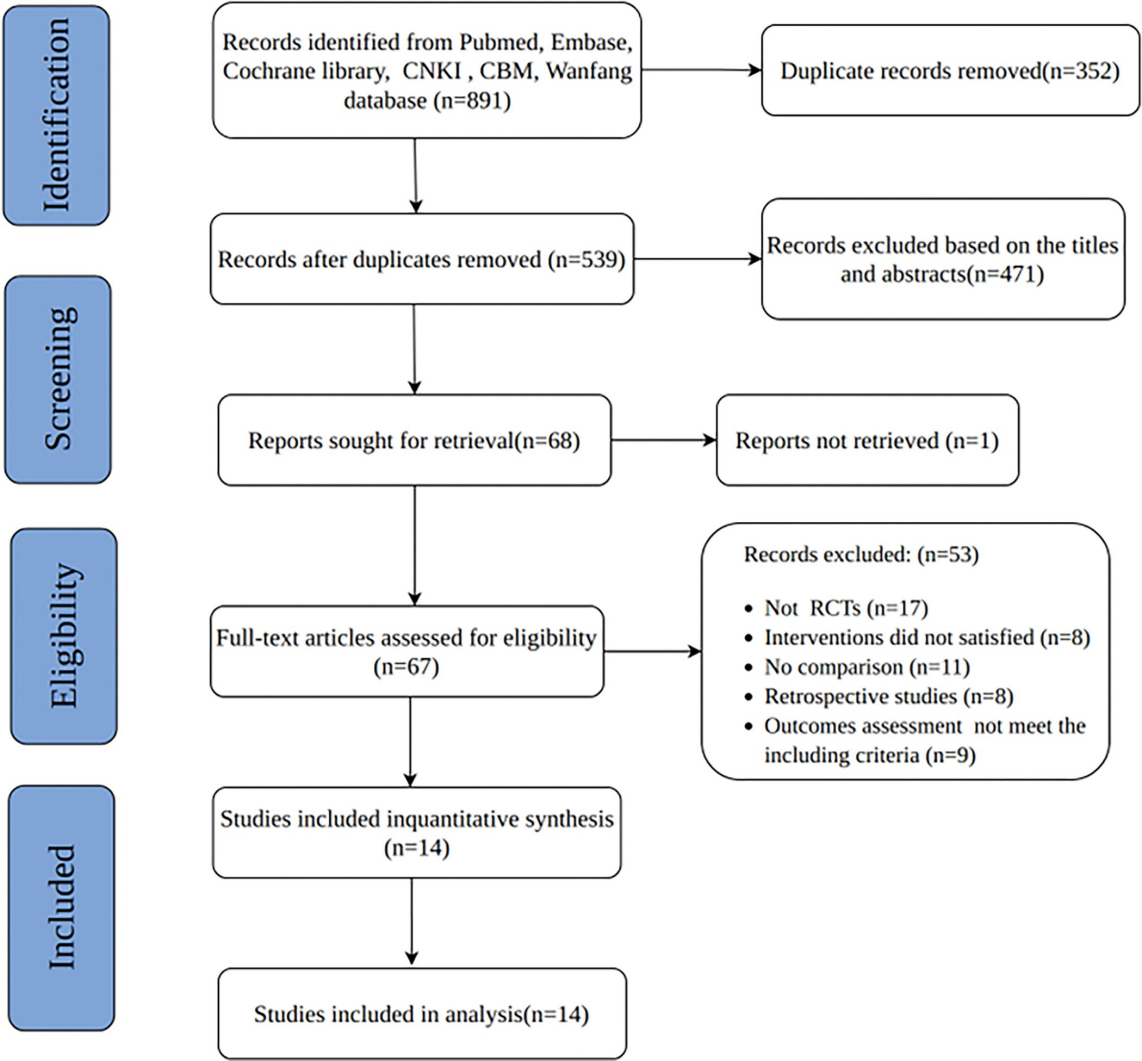
Flowchart of trial selection process for this systematic review.

A comprehensive search of the database from database creation to 28 February 2023 retrieved a total of 891 studies that met the relevant criteria, and 352 duplicates were removed using the literature manager. After reading the titles and abstracts, 471 irrelevant studies were excluded, and 67 literature were selected for full-text reading. 54 of the 67 literature were excluded, and the detailed reasons are shown in the flow chart. Fourteen RCTs were finally included for meta-analysis [18–31]. (Fig 1).

### 3.2 Study characteristics

Detailed characteristics of the included studies including publication year, first author, country, sample size, age, treatment and control group intervention, and treatment period are described in Table 1. The selection of acupoints is shown in Table 2. All studies were conducted and published in China between 2010 and 2022, and a total of 1009 patients with MG were enrolled. With sample capacity ranging from 34 to 154 and therapy duration ranging from 4 weeks to 24 weeks. None of the studies used sham acupuncture as a control. The control group was mainly prednisone or pyridostigmine bromide. Four studies reported a follow-up of 1 to 3 months, while none of the others reported follow-up. Thirteen trials reported effective rate, 4 trials reported TCM relative clinical score, five trials evaluated ACS of MG, and two RCTs reported QMG.

**Table 1:** Characteristics of included studies and patients.

Included studies	Country	Sample size(I/C)	Age [y, mean (SD)] (I/C)	Intervention	Comparison	Treatment period	Follow-up	Adverse events	Outcomes
Zhou et al., 2020 [18]	China	42 / 42	45.25 (10.21) / 46.85 (10.52)	Acupuncture, Bupi Yi shen decoction, Prednisone, 5-10mg/(kg. day)	Prednisone, 5-10mg/(kg. day)	6weeks	3 months	Not report	**①②⑥⑦⑧⑨**
Zhao et al., 2016 [19]	China	41 / 41	35 (7) / 35 (6)	Acupuncture	Pyridostigmine bromide, 30~60 mg, tid.	6weeks	Not report	Not report	**①**
Zhou et al., 2016 [20]	China	29 / 28	36.67 (15.04) / 34.80 (14.03)	Acupuncture, Sheng Siwu decoction.	Pyridostigmine bromide, 30~60 mg, tid.	8weeks	Not report	Not report	**①②④**
Zhang et al., 2012 [21]	China	18 / 16	26.89 (2.58) / 27.69 (2.33)	Acupuncture, Pyridostigmine bromide, 30~60 mg, tid.	Pyridostigmine bromide, 30~60 mg, tid.	12weeks	Not report	Not report	**①④**
Yang et al., 2017 [22]	China	30 / 30	31.63 (5.43) / 30.58 (5.48)	Acupuncture, Shengyang Jutuo decoction	Pyridostigmine bromide, 30~120 mg, qd.	4weeks	3 months	Not report	**①**
Zhang et al., 2019 [23]	China	29 / 29	40 (14) / 41 (13)	Acupuncture, Bupi Yiqi decoction.	Pyridostigmine bromide, 30~120 mg, qd.	8weeks	Not report	Not report	**①**
Wu et al., 2010 [24]	China	20 / 20	30.36 (12.66) / 32.63 (18.21)	Acupuncture. Pyridostigmine bromide, 240~480 mg, tid.	Pyridostigmine bromide, 240~480 mg, tid. Prednisone, 60~100mg, tid.	4weeks	1 months	Not report	**①**
Sheng et al., 2015 [25]	China	35 / 35	34 (11) /39 (10)	Acupuncture, Qiangli Yiqi decoction.	Pyridostigmine bromide, 60 mg, tid.	6weeks	3 months	Not report	**①②④**
Meng et al., 2020 [26]	China	77 / 77	2.1 (0.9) / 2.2 (1.1)	Acupuncture	Pyridostigmine bromide, 20 mg, tid.	Not report	Not report	Not report	**①**
Hu et al., 2021 [27]	China	32 / 31	48.21 (8.47) / 49.84 (7.21)	Acupuncture, Buzhong Yiqi decoction.	Pyridostigmine bromide, 60 mg, tid.	4weeks	Not report	Not report	**①②**
He et al., 2013 [28]	China	33 / 33	41.5 (8.6) / 40.2 (7.2)	Acupuncture, Pyridostigmine bromide, 60 mg, tid.	Pyridostigmine bromide, 60 mg, tid.	Not report	Not report	Not report	**①⑤**
Fang et al., 2018 [29]	China	72 / 72	25.6 (7.2) / 25.9 (7.2)	Acupuncture, Shenyang Jutuo decoction.	Polyvinyl alcohol eye drops	4weeks	Not report	Not report	**①**
Jiang et al., 2015 [30]	China	20 / 20	49.33 (12.85) / 47.07 (11.20)	Acupuncture, Pyridostigmine bromide, 60 mg, tid.	Pyridostigmine bromide, 60 mg, tid.	24weeks	Not report	Not report	**①③④⑤**
Xu et al., 2022 [31]	China	29 / 28	40 (14) /41 (13)	Acupuncture, Pyridostigmine bromide, 60 mg, tid.	Pyridostigmine bromide, 60 mg, tid.	8weeks	Not report	Not report	**④**

T, intervention group; C, comparison group; tid, three times a day; qd, one time a day; ①, effective rate; ②, TCM relative clinical score, Traditional Chinese Medicine relative clinical score; ③,Quality of life; ④, ACS of myasthenia gravis, Absolute clinical score of myasthenia gravis; ⑤, QMG, Quantitive myasthenia gravis score. ⑥, sIL-6R. ⑦, CD3^+^ (%). ⑧, CD4^+^ (%). ⑨, CD4^+^/ CD8^+^

**Table 2:** Formulas of acupoint selection.

References	Style	Main acupoints
Zhou et al., 2020 [18]	WA	Nei-guan (PC6), Zu-san-li (ST36), Huan-tiao (GB30), Meng-men (DU4), Xuan-zhong (GB39), San-yin-jiao (SP6), He-gu (L14), Da-chang-yu, Shou-san-li (LI10), Yang-ling (GB34), Wei-zhong (BL40).
Zhao et al., 2016 [19]	WA	Wei-zhong (BL40), Da-chang-shu, Meng-men (DU4), Shen-shu, Huan-tiao(GB30), Nei-guan (PC6), San-yin-jiao (SP6), Shou-san-li (LI10), He-gu (L14).
Zhou et al., 2016 [20]	MA	Bai-hui (GV20), Shang-xing, Yang-bai (LR9), Tai-chong (GB9), Si-zhu-kong, Yu-yao, Cuan-zhu, Wai-guan, Zu-san-li (ST36), San-yin-jiao (SP6), Tai-chong (LR3)
Zhang et al., 2012 [21]	MA	Bai-hui (GV20), Wei-zhong (BL40), Da-chang-shu, Meng-men (DU4), Shen-shu, Huan-tiao(GB30), Nei-guan (PC6), San-yin-jiao (SP6), Shou-san-li (LI10), He-gu (L14).
Yang et al., 2017 [22]	MA	Yu-yao, Zhong-zhu, Guang-ming (GB37), Shou-hai (K16), Bai-hui (GV20), Zu-san-li (ST36), Shen-mai (BL62)
Zhang et al., 2019 [23]	MA	Yu-yao, Zhong-zhu, Guang-ming (GB37), Shou-hai (K16), Bai-hui (GV20), Zu-san-li (ST36), Shen-mai (BL62), Shou-san-li (LI10), He-gu (L14).
Wu et al., 2010 [24]	MA	Tan-zhong, Shi-men (RN5), Guang-yuan (RN4), Zhong-wan (RN12), Yang-ling-quan (GB34), Xuan-zhong (GB39), Zu-san-li (ST36), Tai-Chong (LR3)
Sheng et al., 2015 [25]	MA	Zu-san-li (ST36), Xue-hai (SP10), Feng-long, San-yin-jiao (SP6), Qu-chi (LI11), He-gu (L14).
Meng et al., 2020 [26]	MA	San-yin-jiao (SP6), He-gu (L14), Zu-san-li (ST36), Shou-san-li (LI10).
Hu et al., 2021 [27]	MA	Zu-san-li (ST36), Tai-chong (GB9), He-gu (L14), Tai-yang, Feng-Chi (GB20), Yang-Bai (GB14), Si-bai, Zan-zhu
He et al., 2013 [28]	MA	He-gu (L14), Nei-guan (PC6), Guang-ming (GB37), Shou-san-li (LI10), Zu-san-li (ST36), Shen-shu, Bai-hui (GV20).
Fang et al., 2018 [29]	MA	Shi-men (RN5), Guang-yuan (RN4), Zhong-wan (RN12), Yang-ling-quan (GB34), Xuan-zhong (GB39), Zu-san-li (ST36), He-gu (L14)
Jiang et al., 2015 [30]	MA	Bai-hui (GV20), Shen-ting (DU24), Yin-tang, Shui-gou (DU26), Nei-guan (PC6), Shen-men (HT7), Feng-chi (GB20), Tian-zhu (BL10), Jian-yu, Wei-zhong (BL40), Yin-ling-quan (SP9), Zu-san-li (ST36), Shen-shu, Ming-men (DU4)
Xu et al., 2022 [31]	MA	Bai-hui (GV20), Wei-zhong (BL40), Da-chang-shu, Meng-men (DU4), Shen-shu, Huan-tiao(GB30), Nei-guan (PC6), San-yin-jiao (SP6), Shou-san-li (LI10),

MA, manual acupuncture; WA, warming acupuncture

### 3.3 Quality assessment of included studies

**Fig 2:**
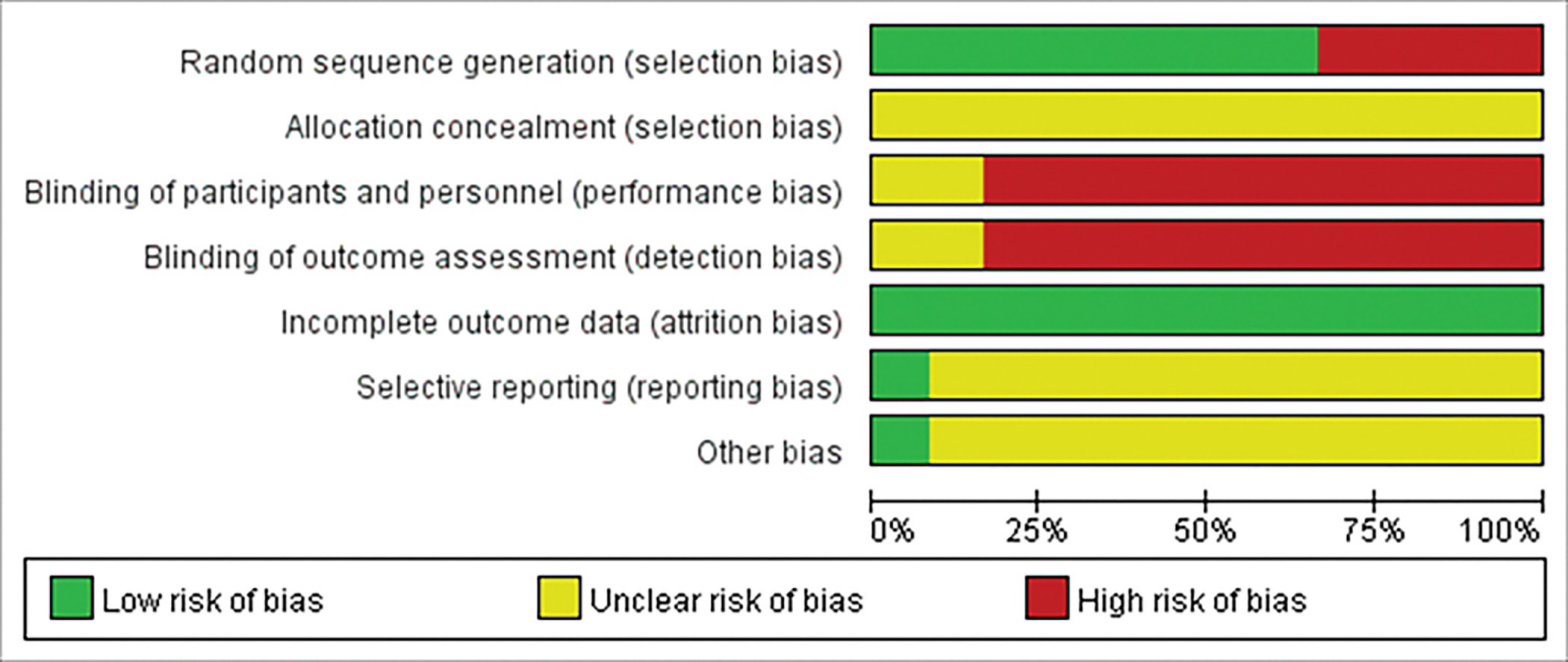
Assessment of risk of bias graph of included studies using the Cochrane tool.

**Fig 3:**
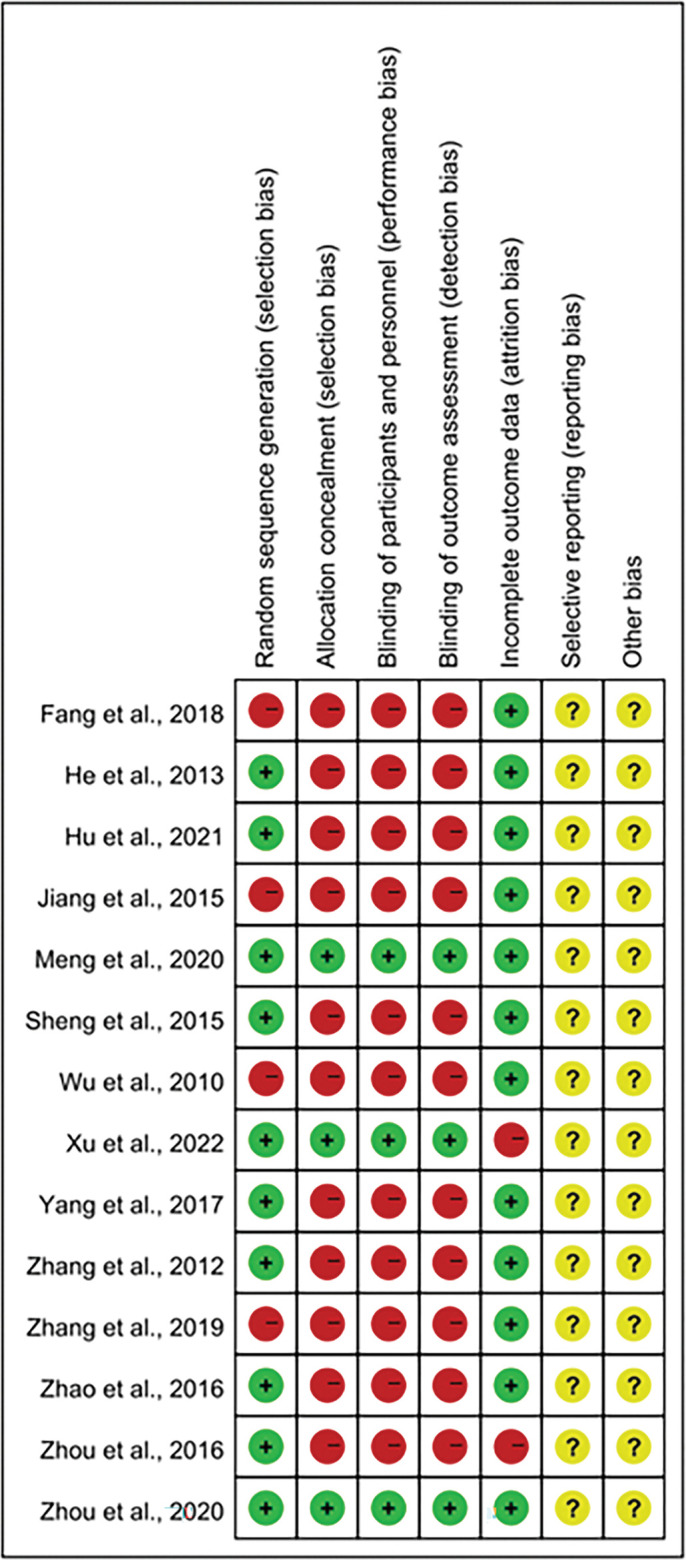
Assessment of risk of bias summary of included studies using the Cochrane tool.

Ten studies described in detail the method of generating random sequences, including random number tables, computer-generated random numbers. The remaining four studies did not explain the process. 3 of the 14 studies described the process of allocation concealment. In the remaining studies, the allocation concealment and blinding processes were not clearly described. Most of the included RCTs were considered to be at “high risk” for performance bias and detection bias. Because 2 RCTs had missing patients, we conclude that both trials were at “high risk” for incomplete outcome data. Moreover, 12 RCTs had a sample size of less than 100, and eight RCTs did not report follow-up. The overall quality of the RCTs was not high, as most RCTs were rated as “unclear” and “high risk”. Clear details of the methodological quality assessment are provided in Figs 2 and 3.

### 3.4 Meta-analysis results

#### 3.4.1 Effective rate

**Fig 4:**
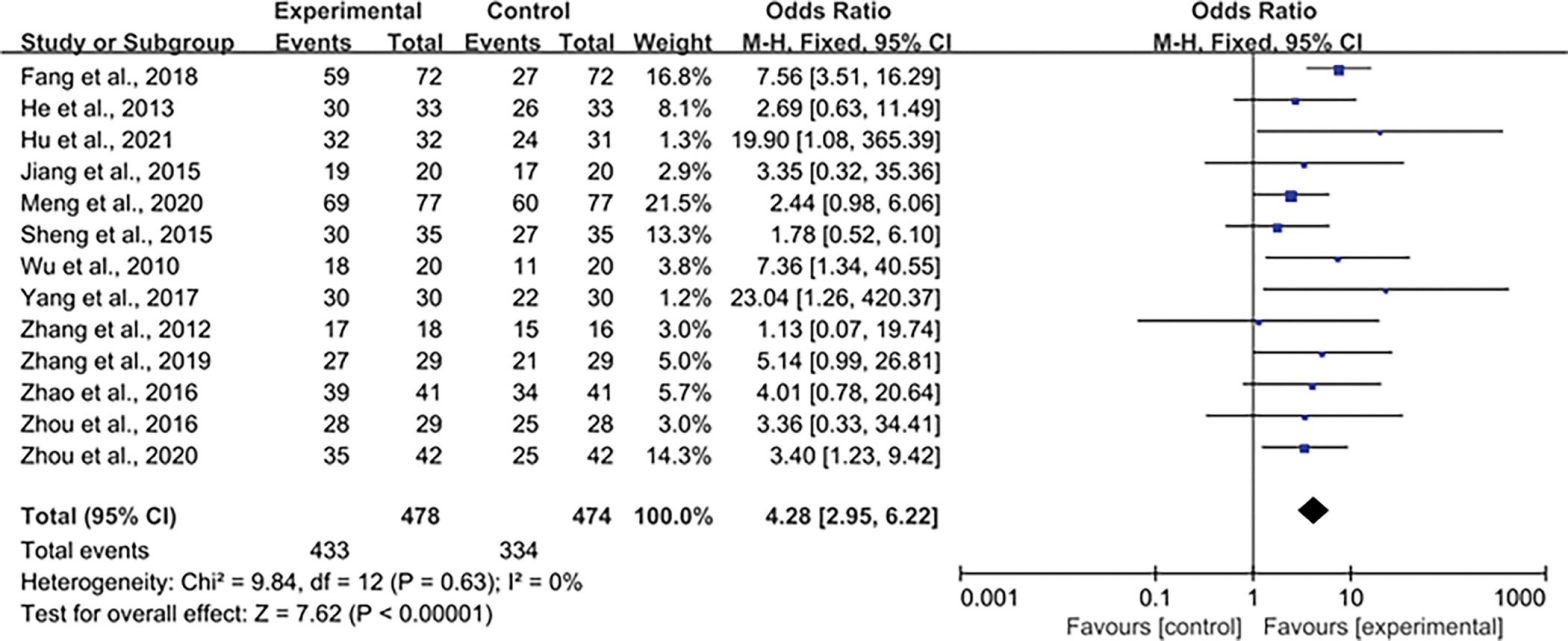
Forest plot of the effective rate for myasthenia gravis.

13 included studies involving 952 patients reported effective rate. There was no statistical heterogeneity among the studies (P = 0.63, I^2^ = 0%), and the fixed effect model was used for analysis (Fig 4). This results of Meta-analysis showed that the effective rate of the experimental group was higher than that of the control group [OR = 4.28, 95% CI = (2.95, 6.22) ], and the difference was statistically significant (P < 0.001).

#### 3.4.2 Traditional Chinese Medicine (TCM) relative clinical score

**Fig 5:**
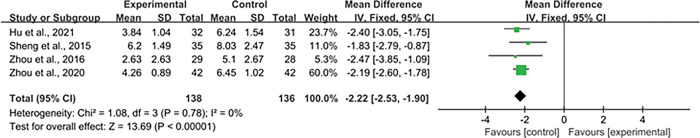
Forest plot of the TCM relative clinical score for myasthenia gravis; outcome: TCM relative clinical score; TCM, Traditional Chinese Medicine.

A total of four RCTs were included with 247 patients assessed TCM relative clinical score. The heterogeneity among the studies was mild (P = 0.78, I^2^ = 0%), and the fixed effect model was used. The results of meta-analysis showed that the TCM relative clinical score of the experimental group was lower than the control group [WMD = -2.22, 95% CI (-2.53, -1.90) ], and the difference was statistically significant (P < 0.001) (Fig 5).

#### 3.4.3 ACS of myasthenia gravis

**Fig 6:**
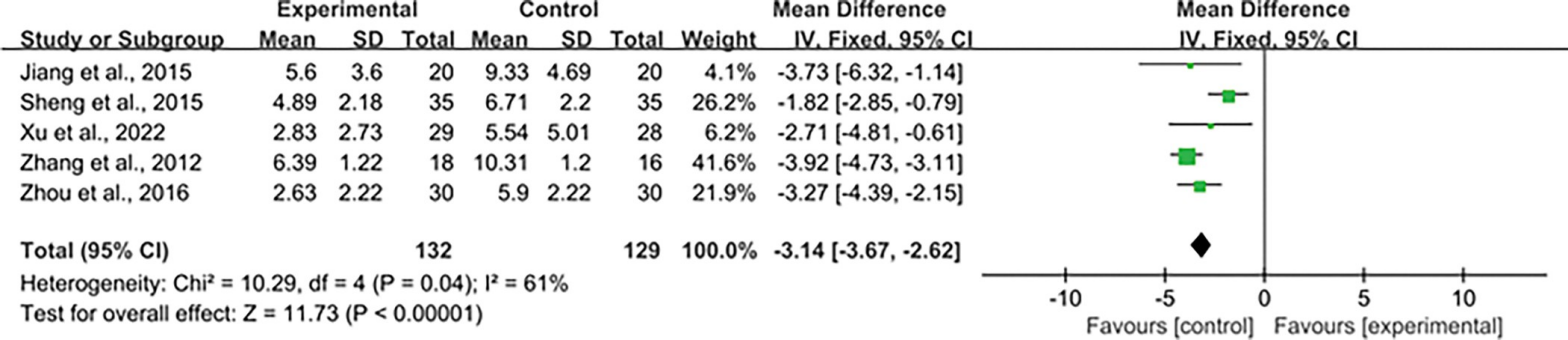
Forest plot of the ACS of myasthenia gravis; ACS, absolute clinical score.

A total of five studies evaluated the ACS of myasthenia gravis. There was significant heterogeneity between studies (P = 0.004, I^2^ = 61%), random effect model was used for analysis.The results of Meta-analysis revealed that the ACS of myasthenia gravis in the experimental group was significantly lower than the control group [WMD = -3.14, 95% CI = (-3.67, -2.62)], and the difference was statistically significant (P < 0.001), suggesting that the experimental group was superior to the control group in improving the clinical symptoms of myasthenia gravis (Fig 6).

#### 3.4.4 Quantitive myasthenia gravis score

**Fig 7:**
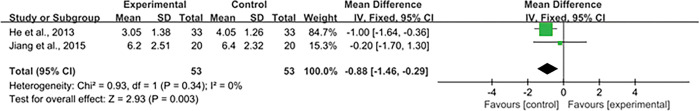
Forest plot of the QMG score; QMG score, Quantitive myasthenia gravis score.

Only 2 trails evaluated quantitive myasthenia gravis score (QMG). This results of Meta-analysis showed that QMG of the experimental group was lower than that of the control group [WMD = -0.88, 95% CI = (-1.46, -0.29), P < 0.001] with mild heterogeneity (P = 0.34, I^2^ = 0%) (Fig 7).

#### 3.4.5 Quality of life and adverse events

Two RCTs involving 106 MG patients evaluated the quality of life. The SF-36 Questionnaire was used to investigate the quality of life of MG patients in one study. Another study used the Busch Modified Quality of Life Scale. The research results suggested that acupuncture as an auxiliary means has a positive effect in improving the quality of life. Only one study mentioned adverse events, that is, there were no obvious adverse events between the two groups.

### 3.5 Publication bias

**Fig 8:**
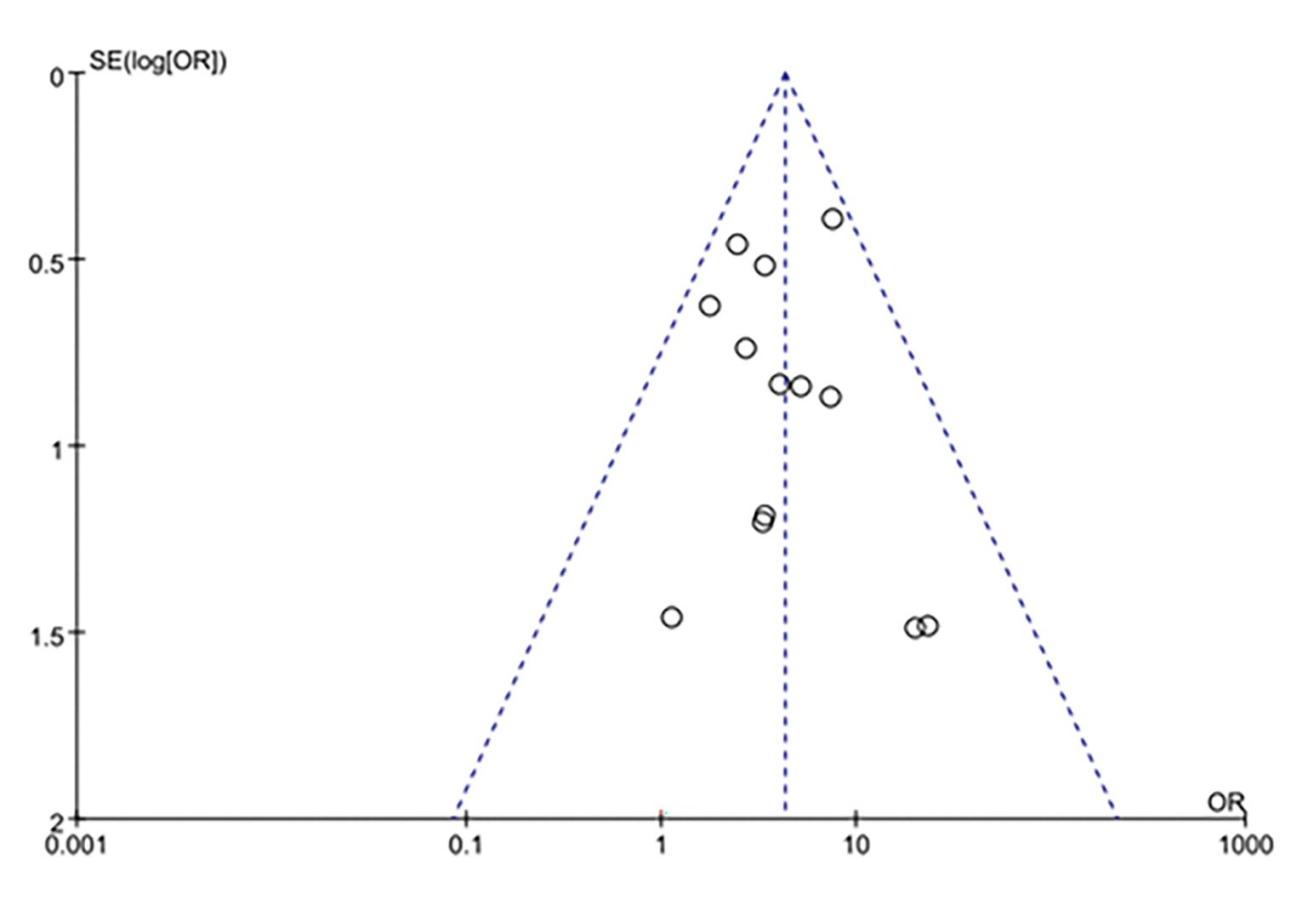
Funnel plots of included studies.

The effective rate from more than 10 studies were assessed for publication bias, and publication bias assessments are presented as funnel plots (Fig 8). From the roughly symmetrical shapes of these funnel plots, no obvious publication bias was observed.

## 4. Discussion

In this meta-analysis on acupuncture treatment for MG, a total of 14 relevant literatures were included, and the included subjects were all patients who met the diagnostic criteria of relevant MG. A total of 1009 patients were enrolled in the analysis, including 507 patients in the experiment group and 502 patients in the control group. The main outcome indicator included in this meta-analysis was the effective rate. The secondary outcome indicator were TCM relative clinical score, ACS, QMG.The literature on adverse reactions and recurrence rates after treatment included in this study is less described, and the possibility of selective reporting by researchers cannot be ruled out. Considering the limited number of literature included in this meta-analysis, and the concept of acupuncture treatment is broadly, there are other clinical operation methods involving the concept of acupuncture treatment that have not been included, such as acupoint direct current stimulation, acupoint injection, auricular point sticking, auricular magnetic stimulation and other programs have not been reflected [32]. This meta analysis results suggested that acupuncture or acupuncture combined drugs can improve the effective rate of MG treatment [OR = 4.28, 95% CI = (2.95, 6.22), P < 0.001], and can also reduce TCM relative clinical score [WMD = -2.22, 95% CI (-2.53, -1.90), P < 0.001], ACS [WMD = -3.14, 95% CI = (-3.67, -2.62), P < 0.001], and QMG [WMD = -0.88, 95% CI = (-1.46, -0.29), P < 0.001]. It has been found that acupuncture improves immune function by regulating peripheral blood lymphocytes and neutrophils biphasically and improving the patient’s T-lymphocyte subsets and natural killer cells [33]. In addition, modern studies have shown that acupuncture can promote the functional activity of acetylcholine [24].

The comprehensive demonstrated that acupuncture as auxiliary has a positive effect on treatment of MG. However, the effect of acupuncture on improving quality of life is unclear. Only 2 studies assessed quality of life and did not use the same scale. Most studies have focused on the effectiveness of acupuncture, and little attention has been paid to the side effects. There is uncertainty about the adverse events of acupuncture due to insufficient attention to adverse events in RCTs.

Through searching databases, Jin et al. found high- frequency acupoints for the treatment of MG were Zu-san-li (ST36), Yang-bai (GB14), Cuan-zhu (BL2), and Yu-yao. Zu-san-li (ST36)—Cuan-zhu (BL2) combination was preferred. The mechanisms of acupuncture in the treatment of MG are mainly as follows: (1) Acupuncture can reduce the expression of Forkhead Box Class O3a (FOXO3a) transcription factors in atrophic skeletal muscle tissue. By inhibiting the activation of FOXO3a, it can reduce the expression of Muscle Atrophy F-box (MAFbx) and Muscle Ring Finger-1 (MuRF1) mRNA, thereby preventing the degradation of muscle proteins, increasing the cross-sectional area of muscle fibers, and improving the degree of skeletal muscle atrophy; [34] (2) Acupuncture can improve the level of mitochondrial fission and fusion. By increasing the expression of fusion protein Optic Atrophy 1 (Opa1) and fission protein Dynamin-related Protein 1 (Drp1), a new balance between mitochondrial fusion and fission is established. This balance can promote the synthesis and function of mitochondrial ATP, thereby enhancing muscle strength; [35] (3) Acupuncture can excite the cholinergic N receptor and activate the cholinergic anti-inflammatory pathway (CAP), thereby exerting an anti-inflammatory effect and delaying the progression of myasthenia.

The present study suggested that acupuncture may be beneficial for patients with MG and provides a new treatment for the clinical practice of MG. The advantages of traditional medicine and modern medicine can complement each other to help patients rebuild their immune function and reduce adverse reactions and recurrence rates. However, the sample size of RCTs included in this study was small and all were from China, and the effectiveness, safety, durability, and acceptability of acupuncture for MG hinder its widespread use in the treatment of MG. We look forward to higher quality RCTs, including placebo-controlled trials, larger sample quantity, more final indicators, and longer follow-up duration to evaluate clinical significance and safety. In addition, attention should be paid to adverse events in RCTs in the future. More animal acupuncture trials in the future are also needed to further validate and clarify the underlying mechanisms of acupuncture for MG.

This study has the following limitations: (1) In terms of literature quality, the quality of the included pieces of literature are generally low, all of which are in Chinese which may lead to a language bias and limit the generalizability of the findings, most of which not mentions blinding of outcome assessment use; (2) Intervention measures on the one hand, the drug dosage, treatment time, and follow-up time of individual studies are inconsistent, leading to differences in observation results and reducing the strength of evidence for conclusions; (3) In terms of outcome indicators, there are few literatures that include absolute clinical scores and TCM relative clinical score for MG. The included articles mentioned that adverse reactions and recurrence rates were less described, and selective reporting cannot be ruled out; (4) In terms of follow-up period, only four RCTs mentioned follow-up periods ranged 1 months to 3 months. In order to improve the evidence strength of the conclusion, more high-quality, large-sample, multi-center clinical randomized controlled trials are needed to comprehensively report the improvement of clinical symptoms, adverse reactions, and immune function indicators of MG, so as to further to verify the efficacy and safety of acupuncture in the treatment of MG.

## 5. Conclusion

This meta-analysis of this study suggests that acupuncture is generally effective as an intervention for MG, providing evidence-based evidence for the use of acupuncture for MG. An integrated approach of acupuncture combined with conventional therapies may provide benefits for MG patients. At the same time, due to the small number of cases included in the literature, the random method of the original literature is not rigorous enough, and the quality of the literature after literature review is relatively low, which will lead to discrepancies between this meta and the literature. These results should be treated with caution.

## Supporting information

S1 Checklist PRISMA 2009 checklist. (DOC)

S1 Table Search strategy. (DOC)

## References

[1] Huda R. Inflammation and autoimmune myasthenia gravis. Front Immunol. 2023;14:1110499. Epub 2023/02/17. doi: 10.3389/fimmu.2023.1110499 ; PubMed Central PMCID: PMC9923104. 36793733 PMC9923104

[2] Farmakidis C, Pasnoor M, Dimachkie MM, Barohn RJ. Treatment of Myasthenia Gravis. Neurol Clin. 2018;36(2):311–37. Epub 2018/04/16. doi: 10.1016/j.ncl.2018.01.011 ; PubMed Central PMCID: PMC6690491. 29655452 PMC6690491

[3] Anderson D, Phan C, Johnston WS, Siddiqi ZA. Rituximab in refractory myasthenia gravis: a prospective, open-label study with long-term follow-up. Ann Clin Transl Neurol. 2016;3(7):552–5. Epub 2016/07/08. doi: 10.1002/acn3.314 ; PubMed Central PMCID: PMC4931720. 27386504 PMC4931720

[4] Mantegazza R, Bernasconi P, Cavalcante P. Myasthenia gravis: from autoantibodies to therapy. Curr Opin Neurol. 2018;31(5):517–25. Epub 2018/08/30. doi: 10.1097/WCO.0000000000000596 . 30156572

[5] Hehir MK, Silvestri NJ. Generalized Myasthenia Gravis: Classification, Clinical Presentation, Natural History, and Epidemiology. Neurol Clin. 2018;36(2):253–60. Epub 2018/04/16. doi: 10.1016/j.ncl.2018.01.002 . 29655448

[6] Deenen JC, Horlings CG, Verschuuren JJ, Verbeek AL, van Engelen BG. The Epidemiology of Neuromuscular Disorders: A Comprehensive Overview of the Literature. J Neuromuscul Dis. 2015;2(1):73–85. Epub 2015/01/01. . 28198707

[7] Gilhus NE, Verschuuren JJ. Myasthenia gravis: subgroup classification and therapeutic strategies. Lancet Neurol. 2015;14(10):1023–36. Epub 2015/09/18. doi: 10.1016/S1474-4422(15)00145-3 . 26376969

[8] Lascano AM, Lalive PH. Update in immunosuppressive therapy of myasthenia gravis. Autoimmun Rev. 2021;20(1):102712. Epub 2020/11/17. doi: 10.1016/j.autrev.2020.102712 . 33197578

[9] Malabaey MAT, Al-Saud AA, Alaska YA, Almas A, Malik A. Pyridostigmine Suicidal Attempt in a Myasthenia Gravis Patient. Am J Case Rep. 2019;20:1418–21. Epub 2019/09/27. doi: 10.12659/AJCR.917308 ; PubMed Central PMCID: PMC6777382. 31554781 PMC6777382

[10] Sieb JP. Myasthenia gravis: an update for the clinician. Clin Exp Immunol. 2014;175(3):408–18. Epub 2013/10/15. doi: 10.1111/cei.12217 ; PubMed Central PMCID: PMC3927901. 24117026 PMC3927901

[11] Wang J, Wong YK, Liao F. What has traditional Chinese medicine delivered for modern medicine? Expert Rev Mol Med. 2018;20:e4. Epub 2018/05/12. doi: 10.1017/erm.2018.3 . 29747718

[12] Acar HV. Acupuncture and related techniques during perioperative period: A literature review. Complement Ther Med. 2016;29:48–55. Epub 2016/12/04. doi: 10.1016/j.ctim.2016.09.013 . 27912957

[13] Wang J, Tian L, Zhang Z, Yuan B, Zhang T, Li X, et al. Scalp-acupuncture for patients with hemiplegic paralysis of acute ischaemic stroke: a randomized controlled clinical trial. J Tradit Chin Med. 2020;40(5):845–54. Epub 2020/10/02. doi: 10.19852/j.cnki.jtcm.2020.05.015 . 33000586

[14] Chavez LM, Huang SS, MacDonald I, Lin JG, Lee YC, Chen YH. Mechanisms of Acupuncture Therapy in Ischemic Stroke Rehabilitation: A Literature Review of Basic Studies. Int J Mol Sci. 2017;18(11). Epub 2017/11/17. doi: 10.3390/ijms18112270 ; PubMed Central PMCID: PMC5713240. 29143805 PMC5713240

[15] Zhang YQ, Jiao RM, Witt CM, Lao L, Liu JP, Thabane L, et al. How to design high quality acupuncture trials-a consensus informed by evidence. Bmj. 2022;376:e067476. Epub 2022/04/01. doi: 10.1136/bmj-2021-067476 . 35354583 PMC8965655

[16] Huang HP, Pan H, Wang HF. "Warming yang and invigorating qi" acupuncture alters acetylcholine receptor expression in the neuromuscular junction of rats with experimental autoimmune myasthenia gravis. Neural Regen Res. 2016;11(3):465–8. Epub 2016/04/30. doi: 10.4103/1673-5374.179060 ; PubMed Central PMCID: PMC4829013. 27127487 PMC4829013

[17] Liberati A, Altman DG, Tetzlaff J, Mulrow C, Gøtzsche PC, Ioannidis JP, et al. The PRISMA statement for reporting systematic reviews and meta-analyses of studies that evaluate health care interventions: explanation and elaboration. PLoS Med. 2009;6(7):e1000100. Epub 2009/07/22. doi: 10.1371/journal.pmed.1000100 . 19621070 PMC2707010

[18] Zhou TT, Zhang Y, Fan Z, Hu Y, Wu CH. Efficacy of Bupi Yishen recipe combined with needle warming moxibustion on myasthenia gravis and its effects on immune function. Shan Xi Traditional Chinese Medicine. 2020;41(11). doi: 10.3969/j.issn.1000-7369.2020.11.039

[19] Zhao G, Hu XL, Zheng JR. Effect of needle warming through moxibustion combined with Tuina on myasthenia gravis. Traditional Chinese Medicine clinical research. 2016;06(0126). doi: 10.3969/j.issn.1674-7860.2016.06.063

[20] Zhou MJ. Clinical Observation on the Treatment of Ocular Myasthenia Gravis with Qi and Blood Deficiency by Modified Shengsiwu Decoction Combined with Acupuncture. Hunan University of Chinese Medicine. 2016.

[21] Zhang D. Clinical Observation on the Treatment of Myasthenia Gravis Type IIA Spleen and Kidney Deficiency Syndrome by Acupuncture Combined with Medicine. Hunan University of Chinese Medicine. 2012.

[22] Yang QK. Observation on the curative effect of Shengyang Jutuo Decoction combined with acupuncture in the treatment of ocular myasthenia gravis. Journal of Practical Traditional Chinese Medicine. 2017;33(6).

[23] Zhang Q, Yu L. Analysis of the Effect of Acupuncture and Moxibustion Combined with Buzhong Yiqi Decoction in Treating Myasthenia Gravis (Chinese). Medicine frontier 2019, 09.

[24] Wu YT, Wang SH, Cui X, Yang LX, Peng ZH, Zheng SH. Effect of warm electroacupuncture on serum IL-6 in patients with myasthenia gravis. Contemporary Medicine. 2010;16(21). doi: 10.3969/j.issn.1009-4393.2010.21.016

[25] Sheng ZY, Chen G, Hu ZH, Yu MQ. Clinical observation of warm needling plus herbal medicine for ocular myasthenia gravis. Shanghai Journal of Acupuncture and Moxibustion. 2015;34. doi: 10.13460/j.issn.1005-0957.2015.06.0540

[26] Meng XY. Analysis of Traditional Chinese Medicine Acupuncture Treatment of Children with Myasthenia Gravis. Longevity magazine. 2020;02(02).

[27] Hu WJ, Du X, Du YM. Clinical observation of electroacupuncture combined with Buzhong Yiqi Decoction in the treatment of ocular myasthenia gravis. Journal of Traditional Chinese Ophthalmology. 2021;31(8). doi: 10.13444/j.cnki.zgzyykzz.2021.08.006

[28] He YJ, Liang ZK, Huang Y, Huang SX. Clinical observation of the traditional Chinese medical science combined with pyridostigmine bromide in treatment of myasthenia gravis. Journal of Qiqihar University of Medicine. 2013;34(14).

[29] Fang XD. Clinical Analysis of Treatment of Eye Myasthenia Gravis with Shengyang Jutuo Decoction Combined with Acupuncture and Moxibustion. Systems Medicine. 2018;3(15). doi: 10.19368/j.cnki.2096-1782.2018.15.146

[30] Jiang A, Li P. Clinical Observation on the Treatment of Myasthenia Gravis by Tongdu Tiaoshen Acupuncture. Sichuan Chinese Medicine. 2015;33(3).

[31] Xu XP, Jiang YB, Guan LH, Ji QJ, Jin Y. Acupuncture combined with western medication for ocular myasthenia gravis: a randomized controlled trial. Zhongguo Zhen Jiu. 2022;42(7):755–9. Epub 2022/07/07. doi: 10.13703/j.0255-2930.20210908-k0003 . 35793884

[32] MacPherson H, Vertosick EA, Foster NE, Lewith G, Linde K, Sherman KJ, et al. The persistence of the effects of acupuncture after a course of treatment: a meta-analysis of patients with chronic pain. Pain. 2017;158(5):784–93. Epub 2016/10/21. doi: 10.1097/j.pain.0000000000000747 ; PubMed Central PMCID: PMC5393924. 27764035 PMC5393924

[33] Jin ZK, Gao B, Zhang LD, Guo ZW, Sun M, Cai RL, et al. Exploration on the diagnosis and treatment of generalized myasthenia gravis with acupuncture and moxibustion based on the study of ancient medical works. Zhongguo Zhen Jiu. 2021;41(7):819–22. Epub 2021/07/15. doi: 10.13703/j.0255-2930.20200727-0004 . 34259419

[34] Chen YF, Jiang XJ, Wang R, Yang ZF, Wu DB, Guo MJ. Electroacupuncture on skeletal muscle and blood of diabetic muscle atrophy rats. Chinese acupuncture and moxibustion. 2020;40(06):629–34. doi: 10.13703/j.0255-2930.20190507–0003 32538015

[35] Corpeno Kalamgi R, Salah H, Gastaldello S, Martinez-Redondo V, Ruas JL, Fury W, et al. Mechano-signalling pathways in an experimental intensive critical illness myopathy model. J Physiol. 2016;594(15):4371–88. Epub 2016/03/19. doi: 10.1113/JP271973 ; PubMed Central PMCID: PMC4967740. 26990577 PMC4967740

