# Supplementary material for: Effects of acupuncture treatment for myasthenia gravis: A systematic review and meta-analysis
Source: PLoS One. 2024 Jan 2;19(1):e0291685. doi: 10.1371/journal.pone.0291685 (PMC10760751; doi:10.1371/journal.pone.0291685)
Supplement: S1 Table — (DOC) [file pone.0291685.s002.doc]

**Table S1**

search strategy

PubMed

#1((((((Myasthenia Gravis, Ocular[Title/Abstract]) OR (Ocular Myasthenia Gravis[Title/Abstract])) OR (Myasthenia Gravis, Generalized[Title/Abstract])) OR (Generalized Myasthenia Gravis[Title/Abstract])) OR (MuSK Myasthenia Gravis[Title/Abstract])) OR (Anti-MuSK Myasthenia Gravis[Title/Abstract])) OR ("Myasthenia Gravis"[Mesh])

#2 ("Acupuncture Therapy"[Mesh]) OR (((((((((((((((((((((((Acupuncture threapy[Title/Abstract]) OR (Acupuncture Treatment[Title/Abstract])) OR (Acupuncture Treatments[Title/Abstract])) OR (Treatment, Acupuncture[Title/Abstract])) OR (Acupuncture therapy[Title/Abstract])) OR (Therapy, Acupuncture[Title/Abstract])) OR (Pharmacoacupuncture Treatment[Title/Abstract])) OR (Treatment, Pharmacoacupuncture[Title/Abstract])) OR (Pharmacoacupuncture Therapy[Title/Abstract])) OR (Therapy, Pharmacoacupuncture[Title/Abstract])) OR (Acupotomy[Title/Abstract])) OR (Acupotomies[Title/Abstract])) OR (electroacupuncture[Title/Abstract])) OR (transcutaneous electrical nerve stimulation[Title/Abstract])) OR (acupoint catgut embedding[Title/Abstract])) OR (auriculotherapy[Title/Abstract])) OR (acupoint injection[Title/Abstract])) OR (fire needle[Title/Abstract])) OR (needle knife[Title/Abstract])) OR (superficial needling[Title/Abstract])) OR (acupressure[Title/Abstract])) OR (cupping jar[Title/Abstract])) OR (moxibustion[Title/Abstract]))

#3 (((randomized controlled trial[Publication Type]) OR (randomized[Title/Abstract])) OR (Controlled clinical trial[Title/Abstract])) OR (Randomly[Title/Abstract])#4 #1 #4 #1 and #2 and #3

Cochrane library

#1 acupuncture therapy:ti,ab,kw

#2 electroacupuncture:ti,ab,kw

#3 fire needle:ti,ab,kw

#4 acupoint catgut embedding:ti,ab,kw

#5 acupuncture treatment:ti,ab,kw

#6 #1 or #2 or #3 or #4 or #5 1

#7 myasthenia gravis:MeSH

#8 myasthenia gravis:ti,ab,kw

#9 ocular myasthenia gravis:ti,ab,kw

#10 generalized myasthenia gravis:ti,ab,kw

#11 MuSK Myasthenia Gravis:ti,ab,kw

#12 Anti-MuSK Myasthenia Gravis:ti,ab,kw

#13 #7 or #8 or #9 or #10 or #11 or #12

#14 clinical trial:ti,ab,kw

#15 randomized controlled trial:ti,ab,kw

#16trials:ti,ab,kw

#17 #14or #15 or #16

#18 #13 and #6 and #17

Embase

#1 'acupuncture therapy'/exp OR 'acupuncture treatment' OR (transcutaneous AND electrical AND ('acupuncture'/exp OR acupoint) AND ('stimulation'/exp OR stimulation)) OR (transcutaneous AND acupoint AND electrical AND stimulation)

OR electroacupuncture

#2 'myasthenia gravis' OR ‘ocular myasthenia gravis’ OR 'generalized myasthenia gravis' OR MuSK Myasthenia Gravis OR Anti-MuSK Myasthenia Gravis

#3 'clinical trial'/exp OR 'clinical trial' OR (('clinical'/exp OR clinical) AND

('trial'/exp OR trial)) OR (randomized AND controlled AND trial) OR trials

#4 #1 AND #2 AND #3

China National Knowledge Infrastructure (CNKI)

( SU='针灸' OR SU='针刺' OR SU='电针' OR SU='头针' OR SU='耳针' OR SU='腹针' OR SU='浮针' OR SU='眼针' OR SU='皮内针' OR SU='火针' OR SU='温针' OR SU='体针' OR SU='腕踝针' OR SU='干针' OR SU='芒针' ) AND (SU='重症肌无力' OR SU='眼肌型肌无力'OR SU='痿症') AND (TKA='随机' OR TKA='对照')

Chinese BioMedical Literature Database (CBM)

(针刺 or 针灸 or 电针 or 头针 or 耳针 or 腹针 or 浮针 or 眼针 or 皮内针 or 火针 or 温针or 体针 or 腕踝针 or 干针 or 芒针) and (眼肌型肌无力 or 全身型肌无力 or重症肌无力) and (随机 or 对照)

Wangfang Database

主题=(针刺 or 针灸 or 电针 or 头针 or 耳针 or 腹针 or 浮针 or 眼针 or 皮内针 or 火针 or 温针or 体针 or 腕踝针 or 干针 or 芒针) and 主题=(眼肌型肌无力 or 全身型肌无力 or重症肌无力) and 主题=(随机 or 对照 )
